# Clary Sage Cultivation and Mycorrhizal Inoculation Influence the Rhizosphere Fungal Community of an Aged Trace-Element Polluted Soil

**DOI:** 10.3390/microorganisms9061333

**Published:** 2021-06-19

**Authors:** Robin Raveau, Anissa Lounès-Hadj Sahraoui, Mohamed Hijri, Joël Fontaine

**Affiliations:** 1 Unité de Chimie Environnementale et Interactions sur le Vivant (UCEIV), Université du Littoral Côte d’Opale, UR 4492, SFR Condorcet FR CNRS 3417, 50 rue Ferdinand Buisson, CEDEX, 62228 Calais, France; robin.raveau@univ-littoral.fr (R.R.); fontaine@univ-littoral.fr (J.F.); 2 Institut de Recherche en Biologie Végétale (IRBV) de l’Université de Montréal, 4101 Rue Sherbrooke E, Montréal, QC H1X2B2, Canada; mohamed.hijri@umontreal.ca; 3 African Genome Center, Mohammed VI Polytechnic University (UM6P), Lot 660, Hay Moulay Rachid, Ben Guerir 43150, Morocco

**Keywords:** trace element-polluted soils, mycobiota, *Salvia sclarea*, arbuscular mycorrhizal fungi

## Abstract

Soil fungal communities play a central role in natural systems and agroecosystems. As such, they have attracted significant research interest. However, the fungal microbiota of aromatic plants, such as clary sage (*Salvia sclarea* L.), remain unexplored. This is especially the case in trace element (TE)-polluted conditions and within the framework of phytomanagement approaches. The presence of high concentrations of TEs in soils can negatively affect not only microbial diversity and community composition but also plant establishment and growth. Hence, the objective of this study is to investigate the soil fungal and arbuscular mycorrhizal fungi (AMF) community composition and their changes over time in TE-polluted soils in the vicinity of a former lead smelter and under the cultivation of clary sage. We used Illumina MiSeq amplicon sequencing to evaluate the effects of in situ clary sage cultivation over two successive years, combined or not with exogenous AMF inoculation, on the rhizospheric soil and root fungal communities. We obtained 1239 and 569 fungal amplicon sequence variants (ASV), respectively, in the rhizospheric soil and roots of *S. sclarea* under TE-polluted conditions. Remarkably, 69 AMF species were detected at our experimental site, belonging to 12 AMF genera. Furthermore, the inoculation treatment significantly shaped the fungal communities in soil and increased the number of AMF ASVs in clary sage roots. In addition, clary sage cultivation over successive years could be one of the explanatory parameters for the inter-annual variation in both fungal and AMF communities in the soil and root biotopes. Our data provide new insights on fungal and AMF communities in the rhizospheric soil and roots of an aromatic plant, clary sage, grown in TE-polluted agricultural soil.

## 1. Introduction

Microbial communities play major roles in soil ecosystems and provide a wide range of services, notably being involved in nutrient biogeochemical cycles, carbon sequestration, and the provisioning of clean air and water [1,2,3,4]. Among soil microbial communities, fungi represent important biomass, providing the key function of organic matter decomposition leading to nutrient cycling and performing crucial roles in humification, mineralization, and soil aggregation processes [5,6]. Hence, fungi play a central role in the functioning and fertility of most natural and managed ecosystems [3,5,6,7]. Some fungi, such as arbuscular mycorrhizal fungi (AMF) belonging to the phylum *Glomeromycota,* are additionally able to form symbiotic associations with more than 80% of terrestrial plants [8]. Being ubiquitous microorganisms in the soil and obligate biotrophs, they contribute to plant health, growth, and productivity and, in that regard, are particularly valuable and well-characterized in agricultural soils [3,9,10]. AMF contribution to agroecosystem functioning has been particularly well-studied [3], as they provide many benefits to their host plant through improved water and mineral supply and can mitigate the adverse effects of biotic and abiotic stresses including soil pollutant damages [10,11,12,13].

Soil pollution by trace elements (TEs) is a worldwide concern due to the major threat they represent, not only to human health, but also to environmental health and ecosystem functioning, due to their non-biodegradability, bioaccumulation, and potent toxicity [6,11]. Notably, TEs can accumulate in cultivated plants, causing growth and productivity issues as well as possible contamination of the food chain, which may be responsible for various disorders and diseases in human beings [11,14,15,16]. It has previously been shown that revegetation of TE-polluted areas could help towards restoring the disturbed ecosystem’s functioning, as well as providing aesthetic value and a reduction in wind and water erosion phenomena [7,17,18]. In that regard, among other potential remediation options, phytomanagement has attracted significant attention. It combines risk-mitigation methods with sustainable and profitable land-uses to reclaim TE-polluted sites. Based on phytotechnologies, it relies on the ability of plants and their associated microorganisms to naturally immobilize inorganic pollutants, such as TEs, in their rhizosphere [18,19,20]. In order for phytomanagement strategies to be an environmentally sustainable and economically viable process, plant biomass valorization is a major component. At present, research is primarily focused on energy crops, such as willow or miscanthus, intended for bioenergy production [21,22,23]. Nonetheless, the cultivation of aromatic plants destined for essential oil (EO) production could represent both an innovative and economically viable alternative for reclaiming TE-polluted areas. Essential oils, as high-added value products, may be valuable in various fields, such as perfumery and cosmetics, medicine, or food industries. As an example, clary sage (*Salvia sclarea* L.) is a xerophytic aromatic plant native to the Mediterranean area, which is able to grow relatively quickly and to develop a sustainable vegetative cover over two or three successive years, thanks to its short-lived perennial life cycle [24,25,26,27]. This Lamiaceae is also able to produce large amounts of biomass, especially in its second cultivation year, resulting in significant EO production, which is valuable to the food and cosmetics industries but is also well known for its antimicrobial and antiseptic properties [24,27,28,29].

The cautious selection of the plant species, based on its ability to grow in conditions that are toxic to most plants, is another key criterion towards successful phytomanagement [18,21]. Therefore, the use of exogenous amendments—either mineral, organic, or microbial—could support plant growth and health. Thus, microbial inoculants, such as AMF or ectomycorrhizal fungi, have previously been evaluated in field trials [30] and were found to improve plant growth, as well as contributing to TEs immobilization, through either precipitation or complexation phenomena occurring in the rhizosphere [11,18,31]. However, the impact of exogenous AMF amendments on the fungal and AMF community compositions, even though it has previously been investigated, remains unclear. Contrasting results have been published. Some reports indicate a clear shaping of the microbial communities in response to AMF inoculation [9,10,32,33,34]; however, some others have demonstrated no influence of inoculation on the native fungal or AMF communities in the rhizosphere [35,36]. For instance, it was described that inoculating with *Glomus* sp. changed the abundance of indigenous AMF and of other fungal members while consistently enriching several bacterial operational taxonomic units [32]. Similarly, inoculating with a *Rhizophagus irregularis* strain resulted in a suppression of the plant roots’ colonization by native AMF taxa and decreased the diversity of the root-colonizing AMF community [9]. AMF affects the soil microorganisms in the rhizosphere of their host plant, leading to the formation of a specific area in the soil called the mycorrhizosphere, as they compete for nutrients and space and exchange complex chemicals to interact with each other [37,38]. In contrast, Ref. [35] demonstrated that inoculation with *Rhizophagus irregularis* DAOM-197198 did not influence either the AMF community diversity in roots or the indigenous AMF community composition. The authors suggest that the indigenous AMF community could be highly abundant and more adapted to both their host plant and the environmental conditions and hence more resilient to external factors such as exogenous inoculation [35].

From the same perspective, even though the short-term effects of TE pollution on microbial communities are well-known, usually leading to a decrease in both richness and abundance of fungal species [13,14,16], the assessment of aged TE pollution in the long-term has been poorly investigated [17]. Furthermore, plant identity has also been demonstrated as a key factor in shaping the microbial communities and, as such, should be carefully evaluated [17,38,39]. In regards to the above, AMF inoculation and plant identity are likely to result in a shift of the fungal and AMF communities in the rhizosphere of the cultivated plant, as well as their respective structuring impact on the communities should be evaluated.

Hence, studying fungal community composition in such conditions could facilitate an understanding of the below-ground ecosystem’s functioning, as well as to potentially identify unique and TE-well-adapted fungal species. From a wider perspective, the identification of fungal species capable of growing in TE-polluted conditions could provide a knowledge basis for the development of remediation methods. Thus, the present study aimed to: (I) Characterize the indigenous fungal and AMF community compositions in TE-aged polluted soil; (II) evaluate and monitor the potential structural changes induced in plant rhizosphere communities by the cultivation of *S. sclarea* over its whole life cycle; (III) assess the effects of the addition of a commercial mycorrhizal inoculum on the native fungal communities, including AMF, over the clary sage life cycle.

## 2. Materials and Methods

### 2.1. Experimental Site

This study was carried out on an aged TE-polluted site (50°25′55.5″ N, 3°02′25.5″ E; altitude 23 m) in the north of France. The area is characterized by a warm, temperate, and humid climate with cool summers. The climatic conditions are marked by average annual precipitation of 742.5 mm and a mean annual temperature of 12 °C, ranging from 3.6 °C in January to 20.3 °C in July (Infoclimat, accessed on 3 March 2020).

The site is a former agricultural field with a size of 1.3 ha. It is located 600 m away from a former Pb and Zn smelter, Metaleurop Nord, whose activities over more than a century generated significant amounts of TE-containing dust, which led to topsoil (0–30 cm) TE pollution [40,41]. The topsoil is characterized by high amounts of Cd, Pb, and Zn, which correspond to concentrations approximately 17-, 11-, and 6-fold higher, respectively, than those reported in regional background levels for agricultural soils [42,43]. The soil texture consists of silt loam with a slightly alkaline water pH (7.9). The soil physicochemical properties were homogenous across the experimental plot. Full descriptions of the experimental site and soil have been provided in [43,44].

### 2.2. Biological Material

Clary sage (*Salvia sclarea* L.), a biennial herbaceous aromatic plant species, was cultivated for two years in the TE-polluted site. Sowing took place in the early spring of 2017, with a sowing density of 300,000 seeds per hectare, using farm-scale equipment. Seeds were provided by ITEIPMAI, a French research institute. A commercial mycorrhizal inoculum, kindly provided by Premier Tech (AGTIV^®^ Specialty Crops, PremierTech, Rivière-du-Loup, QC, Canada) and containing the AMF *Rhizophagus irregularis* (isolate DAOM-197198, 12,000 viable spores per gram of product, 125 g/ha) in a powdery formulation, was also introduced during sowing, mixed directly with sage seeds.

### 2.3. Experimental Design

The experiment was carried out over two successive years (from 2017 to 2018), with an experimental design consisting of two treatments, as described below:

The two-year assessment (repeated-measures design) covered three different monitoring times (namely, the original state of the plot before sowing; the state at first sage harvest, year 1; the state at second sage harvest, year 2) and two treatments: non-inoculated (NI) and inoculated with the mycorrhizal inoculum (I). The surfaces dedicated to each treatment (I or NI, 2000 m² each) were separated from each other by 2 m-wide lanes, which were regularly weeded. The design included five replicates for each condition. It should be noted that the original state of the plot was unvegetated.

### 2.4. Soil and Plant Roots Sampling Procedure

Five soil samples were randomly collected in the early spring of 2017, at the polluted experimental site, following a stratified sampling method to ensure a maximized coverage of the experimental plot in order to assess the site’s original condition before sowing. Sampling was carried out in the 0–30 cm surface horizon, using a manual auger. Two other sampling campaigns, totaling 10 soil samples each, were successively conducted after 21 and 66 weeks of cultivation to respectively assess the state at first and second sage harvests. All the sampling points were geo-referenced. The root systems of the three closest growing plants were collected at each of the ten sampling locations. All samples were collected over a half-day period and transported within 2 h. After sampling, the sage roots were vigorously shaken to remove loosely attached soil (bulk), which was discarded. The careful collection of the rhizospheric soil was done using a sterile brush and spatula tip [45,46]. Sage roots were finally rinsed three more times with sterilized water in order to get rid of the potentially remaining soil particles before DNA extraction. Compositing was carried out using three replicates for each sampling point (either roots or rhizospheric soil). Soil and sage roots were then frozen and stored at −20 °C until further use.

### 2.5. DNA Extractions

#### 2.5.1. Soil Samples

Genomic DNA was extracted for each sampling point in triplicate, directly from 250 mg of soil, using a Nucleospin Soil^®^ kit (Macherey-Nagel, Düren, Germany), according to the manufacturer’s instructions. DNA quality was verified using 1% (w/v) agarose gel and measuring the 260/280 nm and 260/230 nm ratios using a SpectraMax^®^ iD3 device (Molecular Devices LLC, Sunnyvale, CA, USA). The concentration of all samples was determined and DNA extracts were diluted to 25 ng.μL^−1^ for further analyses. The extracted DNA was stored at −20 °C until further use.

#### 2.5.2. Plant Root Samples

The fine roots were kept in Eppendorf tubes and frozen at −20 °C. Genomic DNA from clary sage roots was extracted in triplicate, using a method adapted from [47,48]. Briefly, sage roots were first frozen using liquid nitrogen (−196 °C) in a sterilized mortar and ground into fine powder. Then, 500 mg of the ground roots were subjected to Cetyltrimethylammonium bromide (CTAB, 1.4 M NaCl’ 100 mM Tris-HCl, pH 8.0; 20 mM EDTA, pH 8.0; 2% CTAB), Polyvinylpyrrolidone (PVP 1% *w/v*), β-Mercaptoethanol (5% *v/v*), and activated charcoal (0.5% *w/v*) extraction (30 min; 55 °C). After this incubation period, a centrifugation step was carried out (10 min; 16,000 *g*), and lysate extraction was then performed, in two successive steps, with chlorophorm:isoamylalcohol (24:1). DNA precipitation was performed in the presence of isopropanol (1 h incubation; 25 °C), followed by another centrifugation (10 min; 700 *g*). The DNA pellet was then washed three times in a row, through the addition of ice-cold ethanol (70%), and centrifuged (10 min; 900 *g*) before air-drying at room temperature (approx. 90 min; 20 °C). Finally, the DNA pellet was dissolved in 50 µL of TE buffer (10 mM Tris-HCl, pH 8.0; 1.0 mM EDTA, pH 8.0). The quality assessment of the extracted DNA, normalization to 25 ng.μL^−1^, and storage were performed as described above.

### 2.6. PCR and Sequencing

#### 2.6.1. Fungal ITS

The fungal ITS region was amplified by PCR using a PCR thermal cycler (Surecycler 8800, Agilent Technologies, Les Ulis, France) with CS1 and CS2 barcoded degenerated primers as follows:

Forward primer: **CS1**_ITS_3__Kyo2_F **ACACTGACGACATGGTTCTACA**GATGAAGAACGYAGYRAA, reverse primer: **CS2**_ITS_4__Kyo3_R **TACGGTAGCAGAGACTTGGTCTCT**BTTVCCKCTTCACTCG, with an expected length of 327 bp [49]. CS1 and CS2 adapters with molecular barcodes are displayed in bold.

Triplicate reactions per DNA sample were performed. The reaction mixtures (25 μL) contained 5 μL of Q5 (5X) reaction buffer, 3 µL of Q5 (5X) High GC enhancer, 0.25 μL of Q5^®^ High-Fidelity DNA Polymerase (New England Biolabs France, Evry, France), 0.8 μL of each primer (0.4 µM), 1 μL of dNTPs (0.2 mM), 1 µL of DMSO, 1 µL of Bovine Serum Albumin (BSA; 100 µg.mL^−1^), and 1 ng of DNA template. The PCR conditions were as follows: Initial denaturation at 95 °C for 10 min, followed by thirty-five cycles of 94 °C for 20 s, 47 °C for 30 s, and 72 °C for 20 s, and a final elongation step at 72 °C for 7 min.

#### 2.6.2. 18S rRNA Gene of AMF

As the previously described primers were intended for the assessment of the whole fungal community, the DNA of AMF was specifically amplified using Nested-PCR (Surecycler 8800, Agilent Technologies, Les Ulis, France) in order to obtain an improved resolution of the AMF community composition. The first PCR amplification was performed using a set of primers AML_1_ (ATCAACTTTCGATGGTAGGATAGA) and AML_2_ (GAACCCAAACACTTTGGTTTCC), targeting the small 18S subunit of the rRNA gene, which generates amplicons of about 800 bp [35,50]. The PCR conditions were as follows: Initial denaturation at 94 °C for 3 min, followed by 35 cycles of 94 °C for 1 min, 45 °C for 1 min, and 72 °C for 1 min, and a final elongation step at 72 °C for 5 min. PCR reactions were performed in triplicate, with a reaction volume of 25 µL and conditions as follows: 5 μL of Q5 (5X) reaction buffer, 0.25 μL of Q5^®^ High-Fidelity DNA Polymerase (New England Biolabs France, Evry, France), 0.8 μL of each primer (0.4 µM), 1 μL of dNTPs (0.2 mM), 1 µL of DMSO, 1 µL of BSA (100 µg.mL^−1^), and 1 ng of DNA template.

The second PCR amplification used an identical reaction mixture as described above, including 1 ng of the first amplification as a template, with the set of primers nu-SSU-0595-5-F (**ACACTGACGACATGGTTCTACA**CGGTAATTCCAGCTCCAATAG) and nu-SSU-0948-3-R(**TACGGTAGCAGAGACTTGGTCT**TTGATTAATGAAAACATCCTTGGC) [51], complemented with CS1 and CS2 barcoded adapters (displayed in bold). The reaction conditions were as follows: Initial denaturation at 94 °C for 3 min, followed by thirty-five cycles of 94 °C for 1 min, 58 °C for 1 min, and 72 °C for 1 min, and a final elongation step at 72 °C for 5 min. This second amplification generated an amplicon of about 400 bp in length [35].

#### 2.6.3. Illumina MiSeq Sequencing

The triplicates for each PCR product (*n =* 25 for soil; *n =* 20 for roots) were first indexed and then pooled together and sent for sequencing at the Genome Quebec Innovation Centre (Montreal, QC, Canada) using an Illumina MiSeq producing paired-end reads of 2 × 300 bp in length.

### 2.7. Bioinformatic Processing

Bioinformatic and statistical analyses were performed using the R 3.6.1 software [52]. We should note that a bioinformatic analysis was run separately for each fungal (root or soil) and AMF (root or soil) data set, as these were separately sequenced and, therefore, a specific error model for each data set was calculated. Paired-end sequences for both ITS and 18S rRNA gene data sets were filtered, denoised, dereplicated, and filtered for chimeras using the denoising pipeline DADA2 (v. 1.12), following the described procedure (https://benjjneb.github.io/dada2/tutorial.html, accessed on 14 November 2019). At the end of the workflow, amplicon sequence variants (ASV) were inferred. Due to the highly variable length of the ITS region, which may compromise the reliability of the filtering and trimming steps, a variation of the DADA2 pipeline was used for the ITS dataset, (https://benjjneb.github.io/dada2/ITS_workflow.html, accessed on 20 November 2019). A truncation tool, “Cutadapt” was used for the removal of the ITS primers prior to trimming and filtering with DADA2 [53].

Taxonomic assignment for the ITS data set was performed through the DADA2 pipeline, using the Ribosomal Database Project naïve Bayesian classifier [54]. The Unite fasta release (Unite Community, accessed on 5 November 2019) was used to assign fungal taxa from the kingdom to the genus level. Each fungal ASV was further assigned to functional groups of fungi using FUNguild [55]. For each assignment, a confidence ranking was attributed (“possible”, “probable”, and “highly probable”), reflecting the likelihood that a taxon belongs to a given guild, based on previous peer-reviewed data [55]. Only functional assignments with a probable or higher confidence ranking were taken into account.

For the AMF taxonomy, a two-step workflow was followed. The Silva v132 database formatted for DADA2 [56] was used as a first step to assign fungal taxa from kingdom to genus (minimum bootstrap 80) and compared to the results obtained with a nucleotide BLAST. The first step’s aim was to detect and exclude ASVs identified as non-Glomeromycota at the phylum level. As a second step, a phylogenetic tree was constructed, based on reference sequences from well-identified AMF cultures, in order to support and refine the taxonomic identification of each ASV [51]. Multiple alignment using 86 consensus sequences [57] and the ASVs identified as Glomeromycota was first conducted using Kalign [58]. A maximum-likelihood tree was then calculated using RAxML v8.2.10 [59] through the CIPRES web portal [60], which provides online bioinformatic tools.

Alpha richness (Chao1) and diversity (Shannon) indices were calculated using the phyloseq R package, for both ITS and 18S data sets.

### 2.8. Nucleotide Sequence Accession Number

The ITS and 18S rRNA gene sequences of the whole data set have been deposited in the NCBI Sequence Read Archive database and can be reached under the project accession number PRJNA665726.

### 2.9. Statistical Analysis

Prior to any statistical analysis, the Shapiro and Bartlett tests were conducted to assess the normality and homoscedasticity of the data, respectively. If both conditions were verified, ANOVA analysis was carried out. Otherwise, a Kruskal–Wallis non-parametric test (“kruskal.test” function in R), complemented with a post-hoc Dunn test, were used. The significance of the statistical analyses was considered at α = 0.05.

Rarefaction curves were first created using the “rarecurve” function of the Vegan package in R, in order to verify whether the sequencing depth was adequate and reflected the original diversity or not. Based on Bray–Curtis dissimilarity, permutational multivariate analyses of variance (PERMANOVA) and principal coordinate analyses (PCoA) were conducted with the help of the Vegan package in R. PERMANOVA was run with 1000 permutations using the “adonis” function, with a constrained model for inoculation and time parameters. Venn diagrams were constructed to highlight the numbers of shared ASVs between the experimental conditions with the help of the Vegan package. Correlation heatmaps, based on the obtained Pearson’s correlation matrix, were plotted using the “heatmap2” function of the gplots R package.

## 3. Results

### 3.1. Raw Sequences Bioinformatic Processing

The 25 soil samples and 20 root samples yielded 1,558,856 and 1,403,165 fungal (ITS) raw MiSeq reads for the soil and root biotopes, respectively, while 1,553,389 and 1,193,261 reads were obtained for AMF (18S) from the soil and root biotopes, respectively. After quality filtering and chimera removal, we retained 25,241 and 29,877 fungal sequences for the soil and root biotopes, respectively. From the AMF data, 19,053 and 32,241 sequences remained from the soil and root biotopes, respectively, for community analyses. The complete procedure, with the number of sequences at each step, is shown in Table S1.

To compensate for the uneven sequencing efforts of different samples, a resampling step was performed in order to randomly select the same number of sequences per sample. The results of the rarefaction analyses showed that both data sets (ITS and 18S) tended to reach a saturation plateau (Figure S1). As such, this suggests that the sequencing depth was adequate to capture most of the fungal and AMF diversity from the rhizospheric soil and the roots of clary sage.

### 3.2. Fungal and AMF α-Diversity in Root and Soil Biotopes

The fungal and AMF ASVs richness and α-diversity were evaluated separately for each experimental condition (i.e., culture duration—soil before sowing, year-1, and year-2) and mycorrhizal inoculation (NI or I), and accordingly for root and soil biotopes.

**Figure 1:**
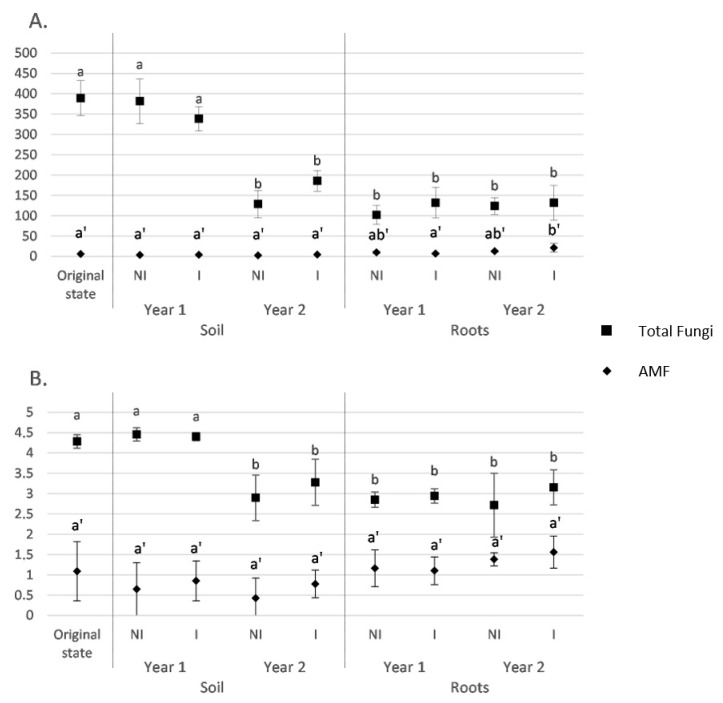
Richness (Chao 1 index, (**A**)) and diversity (Shannon, (**B**)) indices for the five studied conditions for soil whole fungal (ITS) and AMF (18S) communities and the four studied conditions for root whole fungal and AMF communities. Data are means ± SD (*n* = 5) for each condition. Means followed by the same letter do not differ significantly, according to the Kruskal–Wallis test complemented by Dunn post-hoc test (α = 0.05). Lowercase letters and lowercase letters complemented with an apostrophe refer to the test performed for the Total Fungi, and AMF data sets, respectively.NI: non-inoculated; I: inoculated.

According to the Chao1 index, the fungal richness in soil was stable between the soil before sowing and year 1 but significantly decreased between year 1 and year 2 for both NI and I treatments (Figure 1A). No significant difference was observed between I and NI treatments. It is noteworthy that richness measured in roots, regardless of the treatment, were similar to those obtained from soil in year 2 and, hence, were lower than those measured in soil in year 1. In addition, in roots, the fungal richness was found to be equivalent, regardless of the treatment or monitoring time. Regarding AMF richness, the results showed that it was similar in soil, regardless of the treatment or monitoring time. In roots, a significant richness increase between year 1 and year 2 was only observed for the I treatment (*p* < 0.05). However, no significant difference was observed between NI and I treatments. In addition, similar values were obtained in soil and roots, except for the I treatment in year 2.

Regarding the diversity estimator (Shannon index) in soil, our results showed that its value was stable between the soil before sowing and year 1, then dropped between year 1 and year 2 for the total fungi (ITS) data set (Figure 1B). Similar results were observed between NI and I treatments. In roots, the fungal diversity was found to be equivalent to that measured in the soil in year 2, regardless of the treatment or monitoring time. For the AMF data set, the diversity was found to be equivalent, regardless of the biotope (soil or root), the treatment (NI or I), or the monitoring time (soil before sowing, year 1, and year 2). It is noteworthy that the highlighted trends were similar between both total fungi and AMF data sets.

### 3.3. Influence of Mycorrhizal Inoculation and Cultivation Duration on the Fungal Community Composition

**Figure 2:**
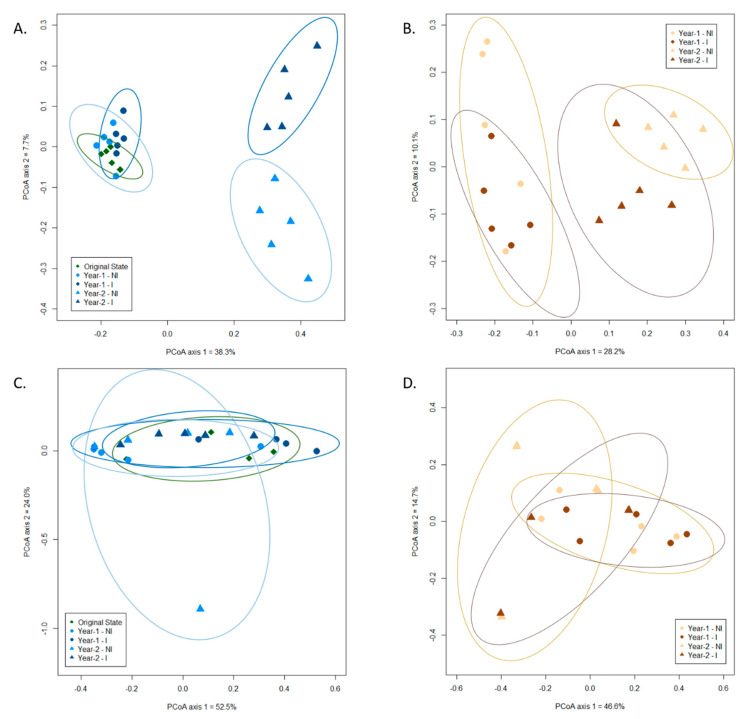
Principal coordinate analysis (scaling 1) based on Bray–Curtis dissimilarity for soil (**A**) and (**C**) and root (**B**) and (**D**) fungal ASVs from the ITS (**A)** and (**B**) and 18S (**C**) and (**D**) data sets in the different experimental conditions. Confidence area of ellipses = 0.95. NI: non-inoculated; I: inoculated.

The effect of the experimental parameters—namely, mycorrhizal inoculation and cultivation duration—on soil and root fungal (ITS and 18S data sets) communities was evaluated through the use of principal coordinate analysis (PCoA), complemented with a Permutational multivariate analysis of variance (PERMANOVA). For the ITS data set, we observed a clear clustering of the fungal communities in response to the cultivation duration in both soil and root biotopes (Figure 2A,B). Specifically, the fungal communities in the soil before sowing and at year 1 were clustered together and separated from those of year-2 (*p* < 0.001). In addition, mycorrhizal inoculation distinguished the fungal communities in year 2 between I and NI treatments (Figure 2A; *p* < 0.05). In roots, fungal communities from year 1 and year 2 were clustered separately, depicting a time effect (Figure 2B). However, no difference was observed between I and NI treatments, regardless of the year. Of the total variance in the data set, the first two principal components together explained 46% of the total soil fungal communities and 38.3% of the total root fungal communities.

These results were confirmed by the PERMANOVA analyses, displaying significant effects of the mycorrhizal inoculation in soil (*p* < 0.05) and of the cultivation duration in both soil and root biotopes (*p* < 0.001 in both cases).

For the 18S data set, the first two principal components of the PCoA ordination together explained 76.5% of the total soil AMF communities and 61.3% of the total root AMF communities (Figure 2C,D). Our PCoA results demonstrated that neither cultivation duration nor mycorrhizal inoculation significantly affected AMF communities in the clary sage rhizospheric soil (Figure 2C) or root biotopes (Figure 2D). Nonetheless, PERMANOVA highlighted a significant effect of the cultivation duration in the root (*p* < 0.01).

For the ITS data set, a total of 761, 696, 644, 206, and 343 fungal ASVs were identified in the soil before sowing, in year 1 (NI and I), and in year 2 (NI and I) treatment samples, respectively, while a total of 221, 294, 214, and 251 fungal ASVs were present in the root biotope for year 1 (NI and I) and year 2 (NI and I) treatments, respectively (Figure S2A,B). The total number of ASVs significantly decreased by about 60% in year 2, regardless of the treatment, depicting a net shift between year 1 and year 2. It is noteworthy that, in year 2, for the I treatment, the drop in the total number of ASVs was lower in comparison with the NI treatment (Figure S2A). In the roots, equivalent numbers of ASVs were shared between I and NI treatments and between the successive years (Figure S2B). However, higher numbers of total ASVs were highlighted for the I treatment, in comparison with the NI one, regardless of the year.

Regarding the 18S data set, a total of 12, 8, 9, 5, and 11 AMF ASVs were identified in the soil before sowing, year 1 (NI and I) treatments, and year 2 (NI and I) treatments, respectively, while a total of 22, 14, 21, and 52 AMF ASVs were present in the roots for year-1 (NI and I) and year-2 (NI and I) treatments, respectively (Figure S2C,D). Due to the relatively low number of AMF ASVs in the soil, there was no clear trend between the different experimental treatments (Figure S2C.). However, in the roots, there was a significant increase (of about 70%) in terms of the number of ASVs for the I treatment between year 1 and year 2 (Figure S2D). This same trend was not observed for the NI treatment between year 1 and year 2. In addition, there was no significant difference in year 1 between NI and I treatments, whereas the total number of AMF ASVs in the I treatment was significantly higher in year 2, in comparison with NI.

### 3.4. Taxonomic Variations in the Fungal Communities in Response to Mycorrhizal Inoculation and Cultivation Time

**Figure 3:**
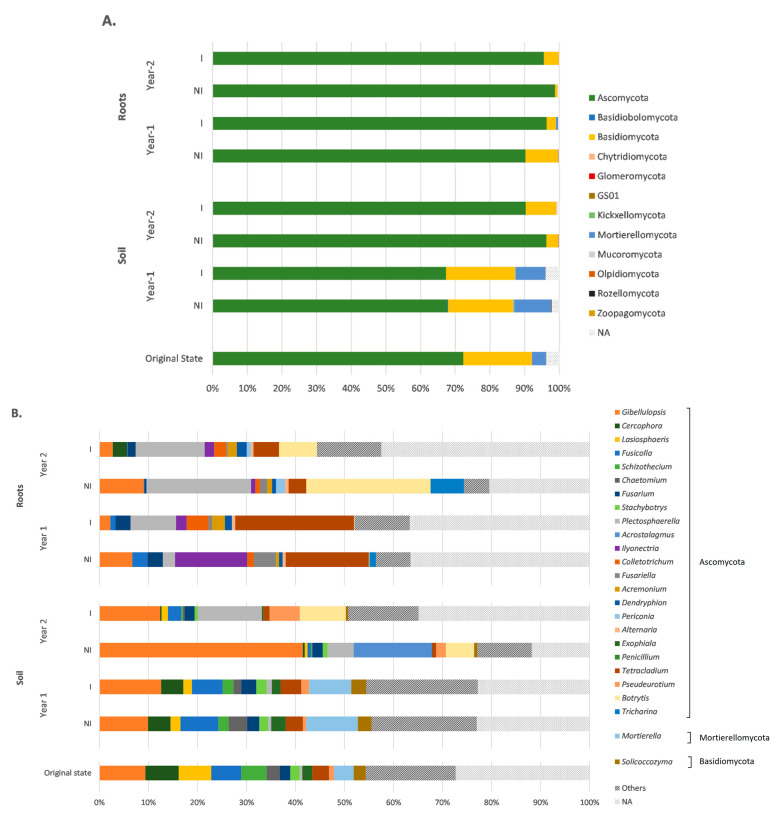
Relative abundances of major phyla (**A**) and genera (**B**) of the fungal ITS rRNA gene data set, according to the different experimental conditions for both root and soil biotopes. The genera were grouped by phylum, and those with a relative abundance lower than 0.5% were gathered in the “Others” group. NI: non-inoculated; I: inoculated; NA: non-assigned.

The fungal diversity and the potential changes induced by the cultivation time and mycorrhizal inoculation were assessed after a taxonomic assignment at phylum, class, order, family, and genus levels. In this regard, soil fungal ASVs were assigned to 12 different phyla, while root fungal ASVs were assigned to four different ones (Figure 3A). Regardless of the experimental condition and biotope, *Ascomycota* was the most-represented phylum. More specifically, it represented about 70% of the total fungal ASVs in the soil before sowing and at year 1, increasing to more than 90% in year 2. *Basidiomycota* and *Mortierellomycota* were also among the most-represented phyla, especially in the soil before sowing and year 1, but decreased in year 2. All the other identified phyla displayed significantly lower relative abundances (below 0.5%), regardless of the condition and the biotope. In roots, the relative abundance of *Ascomycota* exceeded 90%, regardless of the condition. *Basidiomycota* and *Mortierellomycota* were also present in roots but in significantly lower proportions (ranging from 0.005 to 9.4%). In addition to these three phyla, *Glomeromycota* was the only other identified fungal phylum in roots, with a relative abundance of about 0.05%.

At the genus level, the dominant fungal taxa in soil were *Gibellulopsis*, *Mortierella*, *Cercophora*, *Fusicolla*, and *Lasiospheris* (Figure 3B). *Gibellulopsis* was well-represented in all samples, regardless of the experimental condition. This genus was found to be particularly dominant in year 2 in the non-inoculated treatment, with a relative abundance of 42%. In addition, while most of the genera were stable under all treatments and cultivation times, some variations were observed. In particular, *Mortierella* abundance was about 4% in the soil before sowing then increased to 10% in year 1 and dropped to lower than 0.1% in year 2, regardless of the treatment. Furthermore, *Plectosphaerella* and *Botrytis* were poorly represented in the soil before sowing and at year 1, with relative abundances lower than 1%, but significantly increased in year 2 (up to 13%). Finally, *Acrostalagmus* was specifically present in the NI treatment at year 2.

In roots, similar to the soil biotope, *Gibellulopsis* was among the most-represented genera, but along with *Tetracladium*, *Plectosphaerella,* and *Botrytis* (Figure 3B). The proportions of the dominant genera were significantly different, especially between the two sampling times (year 1 and year 2). In particular, *Tetracladium* was extensively represented in year 1 and decreased in year 2, with abundances of 17%, 24%, 4%, and 5% for year 1 and year 2 NI and I treatments, respectively (*p* < 0.05). Conversely, *Plectosphaerella* and *Botrytis* were poorly represented in year 1, but significantly rose in year 2 samples, with respective increases of about 66% and 99% (*p* < 0.01). It should be noted that a relatively high number of fungal ASVs were not assigned to genera, as depicted by the proportion of “Non-Assigned” (“NA”) ranging from 12% to 40%.

### 3.5. Taxonomic Identification of ASVs Belonging to the AMF 18S rRNA Gene Data Set

The results of the phylogenetic analysis (Figure S3) allowed for the assignment of 65 out of the 81 identified ASVs (total AMF data) from the 18S rRNA gene data set to eight genera, namely, *Acaulospora*, *Ambispora*, *Archeospora*, *Claroideoglomus*, *Funneliformis*, *Glomus*, *Rhizophagus*, and *Scutellospora*. Sixteen ASVs could not be assigned at the genus level with a sufficient confidence level, as they did not cluster with any of the consensus sequences. The 65 other ASVs represented six different families (*Acaulosporaceae*, *Ambisporaceae*, *Archeosporaceae*, *Claroideoglomeraceae*, *Gigasporaceae*, and *Glomeraceae*). Notably, 39 (60%) out of the 65 ASVs were assigned to the *Glomeraceae* family and, among them, the genera *Funneliformis* and *Glomus* were the two most-represented ones, with 19 and 17 members, respectively. Only three ASVs belonged to the genus *Rhizophagus*. It is noteworthy that, among the 65 ASVs assigned at the genus level, four of them were present in both root and soil biotopes.

**Figure 4:**
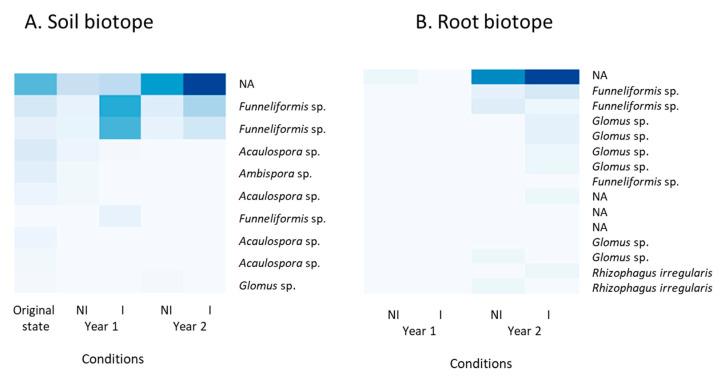
Heatmap representation based on the abundance of the 10 and 15 most-represented ASVs from the 18S rRNA gene data set identified in the rhizospheric soil (**A**) and root (**B**) biotopes, respectively. NI: non-inoculated; I: inoculated; NA: non-assigned.

A heatmap representation was consecutively drawn, based on the relative abundance of the 10 and 15 most-represented AMF ASVs in the rhizospheric soil and the root biotopes, respectively (Figure 4).

This analysis revealed that the most abundant ASV in both soil and roots, which was identical, was present in the initial soil state but significantly decreased in year 1 and, similarly, was scarcely represented in the year 1 root biotope. However, it consistently increased and was mostly represented in both biotopes in year 2. Specifically, in soil, the two most abundant ASVs attributed to the *Funneliformis* genus were particularly abundant in the inoculated treatment, both in year 1 and year 2, but were scarcely represented otherwise. Conversely, ASVs attributed to *Acaulospora* spp. were more abundant in the initial soil state and decreased in year 1 and year 2. *Funneliformis* spp. were also present in roots—specifically, in year 2—with no distinction between I and NI treatments. Furthermore, ASVs identified as *Rhizophagus irregularis* were present in the root biotope in both I and NI treatments in year 2. From an overall perspective, it is noteworthy that ASVs were more abundant in year 2, especially in the inoculated treatment.

### 3.6. Prediction of Functional Assignments of ASVs

**Figure 5:**
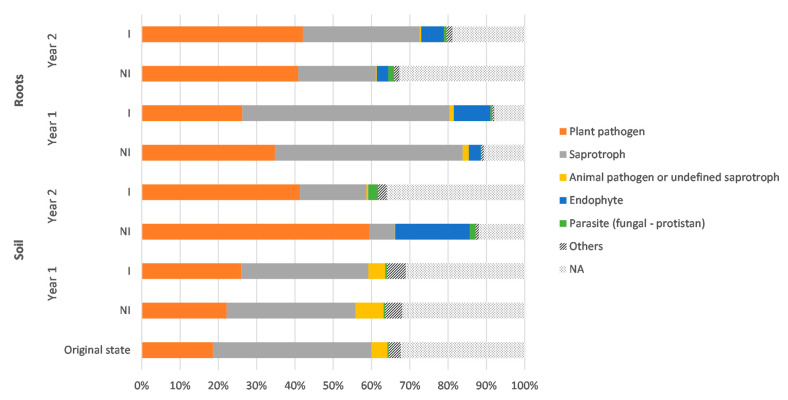
Relative abundances of major fungal functional groups of the ITS rRNA gene data set, according to the different experimental conditions for both root and soil biotopes. The guilds with a relative abundance lower than 0.5% are gathered in the “Others” group. NI: non-inoculated; I: inoculated; NA: non-assigned.

Among the 1239 and 569 fungal ASVs recorded in the soil and root biotopes for the ITS data set, respectively, 48% and 53% were successfully assigned to a functional guild with at least a “probable” confidence ranking, using FUNGuild [55]. This functional assignment revealed a majority of saprotrophic fungi—up to 54% of the total abundance—as well as variable proportions of putative fungal plant pathogens, ranging from 19% up to 60% (Figure 5). It is noteworthy that the relative abundance of plant pathogenic fungi significantly increased in the soil in year 2 (*p* < 0.05) in comparison with the soil before sowing and at year 1; however, in roots, no significant difference was observed between year 1 and year 2. Conversely, the proportions of saprotrophic fungi decreased over time in both biotopes and were at the lowest level during year 2. Besides, endophytic fungi were less represented, especially in soil, with relative abundances close to 0%, except for the NI treatment at year 2; whereas, in roots, the relative abundances ranged from 3%–10%, regardless of the treatment and the monitoring time. In addition, AMF was present but only accounted for less than 0.05% of the total abundance, as only four and five ASVs were assigned to AMF for soil and root biotopes, respectively.

**Figure 6:**
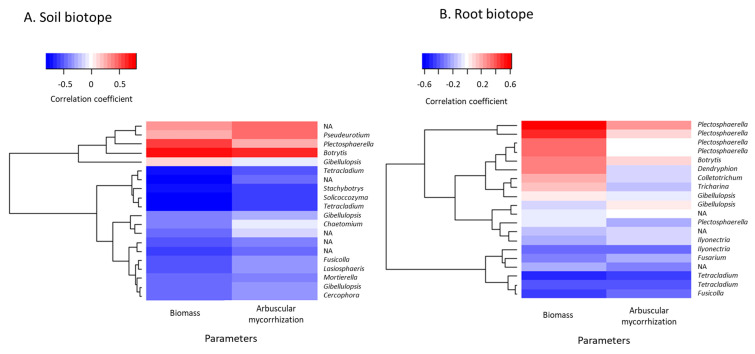
Heatmap representation based on the correlation coefficients between clary sage biomass (dry weight) or arbuscular mycorrhization and the 20 most represented fungal ASVs from the ITS data set in soil (**A**) and root (**B**) biotopes. They respectively belong to 12 and 10 fungal genera. NA: non-assigned.

In addition to the functional assignments, we identified potential correlations between the 20 most abundant ASVs, sage dry weight, and root arbuscular mycorrhizal rates using a heatmap based on Pearson’s correlation coefficients between the 20 most abundant ASVs and sage biomass and root arbuscular mycorrhizal rates were drawn, so as to identify potential correlations between each other, in both the rhizospheric soil and root biotopes (Figure 6).

These correlation coefficients ranged from −0.88 to 0.69 and from −0.55 to 0.63 for the soil and root biotopes, respectively. *Botrytis* was the fungal genus in soil displaying the highest positive correlation coefficients, regarding both sage dry weight and root arbuscular mycorrhizal rates, while *Pseudeurotium* also displayed a high correlation with arbuscular root mycorrhization (Figure 6A). Similarly, in roots, the *Botrytis* genus was strongly and positively correlated to sage biomass, as well as *Plectosphaerella* (Figure 6B). However, regarding arbuscular mycorrhization, the fungal ASVs displayed either low or null correlation coefficients. Conversely, *Tetracladium*, *Stachybotrys,* and *Solicoccozyma* displayed the highest negative correlations in soil, regarding both plant biomass and root mycorrhization, while *Tetracladium* was strongly but negatively correlated to both parameters in the root biotope (Figure 6B).

For the AMF 18S rRNA gene data set, the correlation coefficients ranged from −0.35 to 0.30 and from 0.19 to 0.76 for the soil and root biotopes, respectively. In soil, despite the positive correlation coefficients measured for the most abundant ASVs, no clear correlation was established between the 10 most represented ASVs and plant biomass and arbuscular mycorrhization, given the low values determined for the correlation coefficients (Figure S4). Conversely, in the roots, all the ASVs displayed positive correlation coefficients. In particular, ASVs assigned to *Funneliformis* spp. displayed similar behavior, with respect to plant dry weight, with correlation coefficients equal to or slightly above 0.52, depicting a correlation between each other (Figure S4). Similarly, regarding arbuscular mycorrhization, *Glomus* spp. displayed a positive correlation coefficients with values of about 0.55.

## 4. Discussion

Clary sage has been evaluated as part of a phytomanagement assay aimed at cultivating the aromatic plant on a TE-polluted soil and producing essential oils from the biomass, intended for use as potential biopesticides [44]. However, whereas the impact of this aromatic plant species on the soil bacterial microbiome has been investigated [43], no data are available on its effect on the fungal microbiome. Thus, under field conditions, the effects of clary sage cultivation and mycorrhizal inoculation on the total fungal community composition were determined, with a particular focus on AMF community composition in rhizospheric soil and root biotopes.

### 4.1. Ascomycota Phylum Dominates the Fungal Community in an Aged TE-Polluted Soil before Clary Sage Cultivation

Our results showed that *Ascomycota* and, to a lesser extent, *Basidiomycota* and *Mortierellomycota* were the dominant fungal phyla in our in situ experimental conditions—namely, TE-polluted soil—before clary sage sowing. Notably, *Ascomycota* and *Basidiomycota* were predominant, as they accounted for more than 90% of the relative abundance together. This observation is consistent with recently published studies carried out in both agricultural or aged TE-polluted soils [7,14,17,39,61,62,63]. *Ascomycota* has, in particular, been depicted as the prevalent fungal phylum in various polluted or unpolluted environments due to their diversity in terms of metabolic capacities, owing to the wide variety of enzymes they produce [7,14,63]. They have, for instance, been reported as key decomposers of crop residues in agricultural soils [14,64]. Interestingly, among the members of *Ascomycota* and *Basidiomycota*, many have been shown to display a strong tolerance to TE, even in highly polluted conditions [6,14,62,65]. In these particular environmental conditions, some fungal communities have, indeed, exhibited a capacity to adapt over time and to develop a tolerance to TE [13,17]. In the case of *Ascomycota* and *Basidiomycota*, previous reports have pointed to an induction of the expression of genes coding for metallothionein or phytochelatin synthase, which are involved in fungal cell protection against harmful TE effects [66,67,68]. Regarding the fungal diversity in the TE-polluted site, it appeared similar to (or even slightly higher than) previous results published elsewhere in the literature, considering revegetated or agricultural TE-polluted soils, whatever the richness or diversity index [3,7,16,17,69]. This could notably be explained by the water pH being close to neutral and a soil organic matter content which can be considered as slightly higher than average, in comparison with reference indicators, in our experimental conditions. These parameters have, in fact, been demonstrated as key drivers for the richness and diversity of fungi in soils [6]. Interestingly, 12 ASVs identified as belonging to the phylum *Glomeromycota* and split among four different AMF families were also detected in the TE-polluted soil before clary sage sowing. Notably, a predominance of *Acaulosporaceae* and *Glomeraceae* was found, in accordance with previously published results on TE-polluted soils, which could be explained by their better adaptation to stressful environments, such as excessive amounts of TEs [70,71].

### 4.2. Clary Sage Significantly Shaped the Rhizosphere Fungal Community over its Life Cycle

The results from the PERMANOVA and PCoA analyses indicated the temporal variability of the fungal communities in both rhizospheric soil and root biotopes with high significance (*p* < 0.001) following sage growth. Regarding the diversity indices, similar results were obtained between the soil before sage sowing and the soil sampled before the harvest at year-1. Our results also demonstrated a significant drop, in terms of both richness and diversity, between year 1 and year 2; especially in soil. The same trend was observed for ASV numbers, with a clear decrease between year 1 and year 2. Besides this decrease observed in the rhizospheric soil between year 1 and year 2, a shift should also be highlighted in the roots, depicted by stable richness and diversity indices over time, but which showed changes in terms of relative abundances of fungi. Interestingly, our results suggest that the fungal communities in the rhizospheric soil and in the roots of the cultivated plant tend to become similar over time. Notably, after two years of cultivation, the fungal richness and diversity were found to be equivalent in the rhizospheric soil and in the roots of clary sage, and a similar pattern was observed between the two biotopes at the phylum level, which is consistent with the literature, as it is well-known that fungi associated with the roots are recruited into the surrounding soil community [72]. In a previous related study, the bacterial microbiota of the same aromatic plant was explored [43]. However, this trend was substantially less pronounced in the case of bacterial communities and could suggest that fungal communities are more sensitive to biotic and abiotic factors. Notably, it has previously been reported that organic matter availability was one of the key drivers of the fungal communities’ composition [17]. Furthermore, despite significant differences in relative abundances at the genus level, it appears that the pattern was clearly identical between the state before sowing and at year 1 in soil and differs in year 2, becoming more similar to what was observed in the roots. Notably, several fungal genera, such as *Botrytis* or *Plectosphaerella*, which were very poorly represented in the soil before sowing and at year 1 simultaneously increased in year 2, in both root and rhizospheric soil biotopes, which was expected, as both genera are necrotrophic plant pathogens [73,74].

It has been well-described in the literature that, although the impact of vegetation on soil microbial communities depends on both the growing season and the plant species [10,17,39], fungal diversity usually decreases in the rhizospheric soil, in comparison with the bulk (loosely associated with the roots) or unplanted soil [75]. This fact is notably attributable to the selective pressure exerted by plant root exudates on the fungal communities; even more so in the vicinity of the root, the rhizosphere [17,38,39,63]. First, plant root exudates may support the growth of rhizosphere microbial communities by providing carbohydrates that act as carbon and energy sources for microbial growth [63,76,77]. However, particularly in the case of aromatic plants, such consistent shifts in the fungal communities have been suggested to be bound to the release of various aromatic compounds in their rhizosphere, which exert antifungal activities [2,63,78]. Notably, the involvement of various mono- and sesquiterpenes, such as limonene or menthol, has been postulated [2,63]. This trait has been well-documented for various aromatic plant species, such as *Eucalyptus* spp., *Artemisa annua*, and *Ocimum basilicum*, or from the Lamiaceae family, such as *Mentha arvensis* [2,63,77], but remains unknown for clary sage. Nonetheless, the antifungal activity of clary sage EO and of its major compounds—namely, linalool and linalyl acetate—has repeatedly been demonstrated [28,29,79,80]. In particular, their activity against some fungal genera, which in the present work have been highlighted as significantly decreasing over time, such as *Chaetomium* or *Fusarium*, has previously been established [29,79,80]. Regarding the other fungal genera for which the biggest decreases have been observed, such as *Cercophora* or *Tetracladium*, they are saprotrophic and not phytopathogenic fungi and, as such, might not have been subject to antifungal assessment. One should note that clary sage, as depicted through the previously published results, consistently grows in the second year of cultivation, corresponding to the best harvest year for EO [24,43]. In that regard, the drastic shift observed between year 1 and year 2 could be due to the release of aromatic compounds into the rhizosphere produced in high amounts in that period. Nonetheless, the influence of environmental factors (e.g., soil physico-chemical properties, nutrient availability, and so on) on the fungal community composition has previously been discussed [6,17] and, thus, should not be neglected.

The results of the functional assignment revealed a majority of saprotrophic and plant pathogenic taxa (up to 80%), whereas endophyte and symbiotic taxa were poorly represented in both rhizospheric soil and root biotopes. This goes along with previous findings, reporting a dominance of fungal guilds attributed to pathogens and saprotrophs, accounting for up to 90% of the community [76,81]. On another note, the proportion of saprotrophic taxa decreased over time, replaced by fungal genera known as plant pathogens, such as *Gibellulopsis, Plectosphaerella*, or *Botrytis,* especially between year 1 and year 2. The former was not too surprising, as mono-cropping often results in modifications in the soil microbial abundance and diversity, and usually in an increase in plant pathogenic microorganisms in soil [76,81,82]. Moreover, no fungicidal intervention was carried out during this field trial, which could worsen the previously described traits, as well as potentially increasing the competition between endophytic and pathogenic fungi. Nonetheless, it should be noted that fungal genera pathogenic to plants, such as *Fusarium*, *Gibellulopsis*, or *Botrytis*, even though they have a wide range of host plants, could be harmless for clary sage. Notably, regarding the correlation heatmaps, most of the strong positive correlations between sage biomass or root mycorrhizal rates have been brought to light with potentially phytopathogenic genera, which—to the best of our knowledge—have not been identified as pathogens towards clary sage.

### 4.3. Mycorrhizal Inoculation Shaped the Rhizosphere Fungal Community and Could Favor the Establishment of the Mycorrhizal Symbiosis

Under our experimental conditions, inoculation clearly shaped the soil fungal communities. In fact, a clear clustering in year 2 between I and NI treatments was highlighted by the PERMANOVA analysis (*p* < 0.05) and observed in the PCoA ordination. It, however, did not significantly influence the root fungal community. In the same way, similar diversity and richness indices were determined between I and NI treatments, regardless of the monitoring time and biotope. As previously highlighted, the root exudation of carbon compounds may significantly affect the abundance of soil fungi [39]. Moreover, in the presence of mycorrhizal symbiosis, the host–plant metabolism has been found to be modified, which may result in variations of the root exudation pattern [12,32]. Thus, the stimulation or regulation of specific fungal communities through the release of carbonated or chemical compounds by the plants colonized by AMF is likely to occur and, hence, shape the fungal communities [32,63]. Likewise, the direct release of compounds by the AMF hyphae can result in a shift of the associated fungal communities. Therefore, it not surprising to observe an effect of mycorrhizal inoculation on soil fungal community composition. In particular, some fungal taxa belonging to *Ascomycota*, such as *Nectria* or *Leptosphaeria* genera, were previously established as being associated with AMF mycelia, which could go along with the prevalence of *Ascomycota* observed in our experimental conditions [38,83,84]. It should be noted that in comparison with the bacterial microbiota, where no significant effect of the inoculation was evidenced [43], a higher response of the fungal communities to exogenous mycorrhizal inoculation has been demonstrated in the present work. This goes along with previous studies, highlighting a more pronounced response of the fungal communities to both biotic and abiotic factors [17,85,86].

It is also noteworthy that the decrease, in terms of both ASV number and fungal richness and diversity, observed in soil between year-1 and year-2 was also less pronounced under mycorrhizal inoculation. This may support the idea that, in the presence of the mycorrhizal symbiosis, the host plant and the associated microbial communities could be more resilient regarding both biotic and abiotic stresses [87,88].

In addition, it has previously been suggested that AMF inoculation could result in increased competition between AMF communities (native and exogenous) and could lead to limited efficiency or to a suppression of the mycorrhizal symbiosis establishment and functioning [9,36]. In fact, as has been highlighted in another related publication, the establishment of mycorrhizal symbiosis under the same experimental conditions was successful, while a significant progression of the mycorrhizal rates in response to exogenous AMF inoculation during the second year of cultivation was assessed [43]. However, regarding plant growth in spite of the consistent clary sage growth in TE-polluted conditions, similar results were obtained between I and NI treatments [43]. Given the fact that clary sage is considered a xerophytic plant species and requires low nutrient supplies, its nutrient requirements were probably met in our experimental conditions, considering the soil’s physico-chemical properties [24,89,90,91].

Besides, in our case, no such exclusion of native AMF was visible. In fact, the introduced inoculum contained *R. irregularis,* while the most abundant ASVs present in both the roots and rhizospheric soil of the inoculated plants were identified as belonging to AMF genera such as *Glomus* or *Funneliformis.* In that regard, it is not clear whether the root colonization was due to the presence of the introduced mycorrhizal inoculant or to the native AMF present in the TE-polluted site. In particular, it should be noted that, even though during the second year of cultivation, ASVs identified as *R. irregularis* were detected with low abundance in clary sage roots, it could be a different strain than the inoculated isolate. In addition, the question arises whether the mycorrhizal symbiosis was functional or not, as the clary sage was successfully colonized by AMF. In that regard, monitoring the expression of, for instance, phosphate transporter genes (e.g., *MTPT4*, *LEPT1,* or *STPT3)*, which are often pointed out as markers of symbiosis functioning [92,93,94], could help in addressing this issue.

### 4.4. The AMF Community Changed over Time under Clary Sage Cultivation, but was Not Altered by Exogenous Mycorrhizal Inoculation

It should first be noted that a very limited proportion (<0.5%) of the identified fungal ASVs using the primers targeting the ITS region were attributed to the *Glomeromycota* phylum, which demonstrates that ITS primers are not appropriate for determining AMF diversity. Thus, the use of specific primer pairs targeting the 18S rRNA gene (SSU) appeared to increase the accuracy of AMF species detection and identification.

No significant effect of the mycorrhizal inoculation, as an explanatory parameter of the community composition, was highlighted, regardless of the biotope. A similar result has previously been demonstrated, using the same mycorrhizal inoculant as in the present work under unpolluted field conditions, where the authors suggested a co-evolution of the AMF community in the presence of *R. irregularis* [35]. However, in their experimental conditions, *R. irregularis* was naturally present in the soil, which was not the case in our TE-polluted site. In fact, contrasting results regarding the effects of AMF inoculation on the native AMF community have been brought forward, depicting a context-dependent explanation, particularly in stressful or disturbed conditions [95]. As previously mentioned, in our case, the introduction of the mycorrhizal inoculant did not result in a complete replacement or modification of the native AMF community and, hence, may not have resulted in drastic community changes.

However, interestingly our results demonstrated that clary sage cultivation significantly shaped the AMF community in the plant roots (*p* < 0.01). Our findings also support the same trend as previously reported, namely, the prevalence of the AMF taxa Glomeraceae and a higher number of AMF ASVs identified in roots specific to this biotope [52,96,97]. This was notably explained by a lower AMF biomass in soil—even in the rhizosphere—compared to that in root tissues, depicting the importance of screening both root and soil biotopes [52,97]. Remarkably, 69 distinct AMF ASVs belonging to 12 AMF genera were identified in our conditions, with a prevalence of *Funneliformis* and *Glomus* genera. Hence, in spite of TE pollution and past agricultural practices, which have been demonstrated to be key drivers of the microbial community composition, a relatively high number of AMF species were detected in our TE-polluted site [16,98,99]. This prevalence, in terms of relative abundance of native AMF species, could even suggest a long-term adaptation of these species to the TE-polluted context, which could hence be more suited to surviving and colonizing plant roots in comparison with the exogenous species. Furthermore, while the effects of the AMF community on plant diversity have been well-covered, the reverse effect of plant identity on the AMF community composition and temporal evolution has been less explored [100]. Nonetheless, several reports have pointed out the influence of the host plant on the composition of the AMF community, especially in the roots [100,101,102]. Notably, our results support a strong temporal shift. This trend is in accordance with previous reports, as the AMF ASVs present in the newly germinated seedlings were almost entirely replaced the following year by previously scarcely represented ones [101]. In addition, during the second year of cultivation, the abundance of aromatic compounds in the rhizosphere of clary sage could have an antifungal effect and hence exert selective pressure on the AMF community composition. The trend is similar to that previously observed for the fungal community.

## 5. Conclusions

The main purposes of this field study were to analyze the fungal community composition in the rhizosphere of clary sage cultivated on a TE-polluted soil in the presence of an introduced commercial mycorrhizal inoculum and to monitor their temporal evolution over a two-year cultivation cycle, starting from unvegetated soil.

Our results indicated that both clary sage cultivation and mycorrhizal inoculation significantly shaped the fungal communities in the aromatic plant’s rhizosphere. Notably, it is believed that the cultivation of aromatic plants, such as clary sage, which release root exudates and, in particular, aromatic compounds in their rhizosphere, may be responsible for the temporal evolution of rhizospheric fungal communities, along with environmental and edaphic parameters. Furthermore, besides shaping fungal communities, the introduction of an exogenous mycorrhizal inoculum could favor the in situ colonization of clary sage roots by AMF; however, it was not clear whether the introduction of this inoculum was directly responsible for the root colonization or if it exerted a stimulating effect on the native AMF community. Hence, one of our future prospects is the specific monitoring of the introduced inoculum isolate, as developed by [103].

Our findings also showed the prevalence of *Ascomycota* and *Basidiomycota* in the TE-polluted soil, which have been previously demonstrated to withstand the presence of TE in soils. In addition, given the number of identified AMF species in our TE-polluted site, our data suggest that quite a few AMF species could be further isolated. Notably, if it is well-known that AMF can bear the presence of TE in soils and contribute to their immobilization, their specific response to increasing levels of TE could be relevant to investigating, in view of future potential applications as part of phytomanagement approaches. Further investigations are also required regarding the long-term evolution of the fungal communities. In fact, if the ecosystem functioning may not be affected or in a positive way in response to aromatic plant cultivation and mycorrhizal inoculation, owing to microbial function redundancy, it might also result in important functional losses. Thus, a long-term assessment of structural and functional evolution over time could be a useful tool for assessing and ensuring both the success of phytomanagement and ecosystem functioning.

## Data Availability

The original contributions presented in the study are publicly available.

## Supplementary Materials

The following are available online at https://www.mdpi.com/article/10.3390/microorganisms9061333/s1, Table S1. Descriptive results of Illumina MiSeq sequencing, followed by the step-by-step bioinformatic processing, for the fungal ITS and 18S rRNA genes datasets; Figure S1. Rarefaction curves obtained for the ITS marker (fungi - A. and B.) and for the 18S rRNA gene (AMF - C. and D.), for the soil (A. and C.) and root (C. and D.) biotopes, involving respectively 25 and 20 samples; Figure S2. Venn diagrams showing the overlap of the fungal (ITS dataset – a. and b. and 18S dataset – c. and d.) community overtime for soil (A. and C.) and root (B. and D.) biotopes. These diagrams display the number of total (out of the shapes), specific and shared ASVs. The total numbers of fungal ASVs for the ITS and 18S datasets are respectively 1239 and 20 in soil and 569 and 61 in roots. NI: non-inoculated; I: inoculated; Figure S3. RaxML phylogenetic tree showing the taxonomic assignment of each 18S ASVs to the genus level. The scale represents the branch length corresponding to the expected number of substitutions per site. ASV-R: indicates AMF ASVs identified in the roots; ASV-S: indicates AMF ASVs identified in the rhizospheric soil; Figure S4. Heatmap representation based on the correlation coefficients between clary sage height or arbuscular mycorrhization and the 10 most represented ASVs from the 18S rRNA gene dataset in soil (A.) and root (B.) biotopes respectively. NA: non-assigned taxonomy.
