# Supplementary material for: Clary Sage Cultivation and Mycorrhizal Inoculation Influence the Rhizosphere Fungal Community of an Aged Trace-Element Polluted Soil
Source: Microorganisms. 2021 Jun 19;9(6):1333. doi: 10.3390/microorganisms9061333 (PMC8234821; doi:10.3390/microorganisms9061333)
Supplement: Supplementary file 1 [file microorganisms-09-01333-s001.zip › 1.docx]

**Table S1.** Descriptive results of Illumina MiSeq sequencing, followed by the step-by-step bioinformatic processing, for the fungal ITS and 18S rRNA genes datasets.

|  |  | Fungi | AMF | |  |
| --- | --- | --- | --- | --- | --- |
|  | | Soil | Root | Soil | Root |
| No sequences (sum) | | 1558856 | 1403165 | 1553389 | 1193261 |
| No sequences (mean) | | 70857 | 73851 | 62136 | 62803 |
| No seq. filtered (mean) | | 39754 | 48470 | 50240 | 50838 |
| No seq. merged (mean) | | 26342 | 32428 | 39178 | 46879 |
| No seq. merg. no chimeras (mean) | | 25241 | 29877 | 19053 | 32241 |
| No samples | | 25 | 20 | 25 | 20 |
| No ASV (sum) | | 1239 | 569 | 20 | 61 |
| ASV per sample (mean) | | 279 | 107 | 4 | 13 |


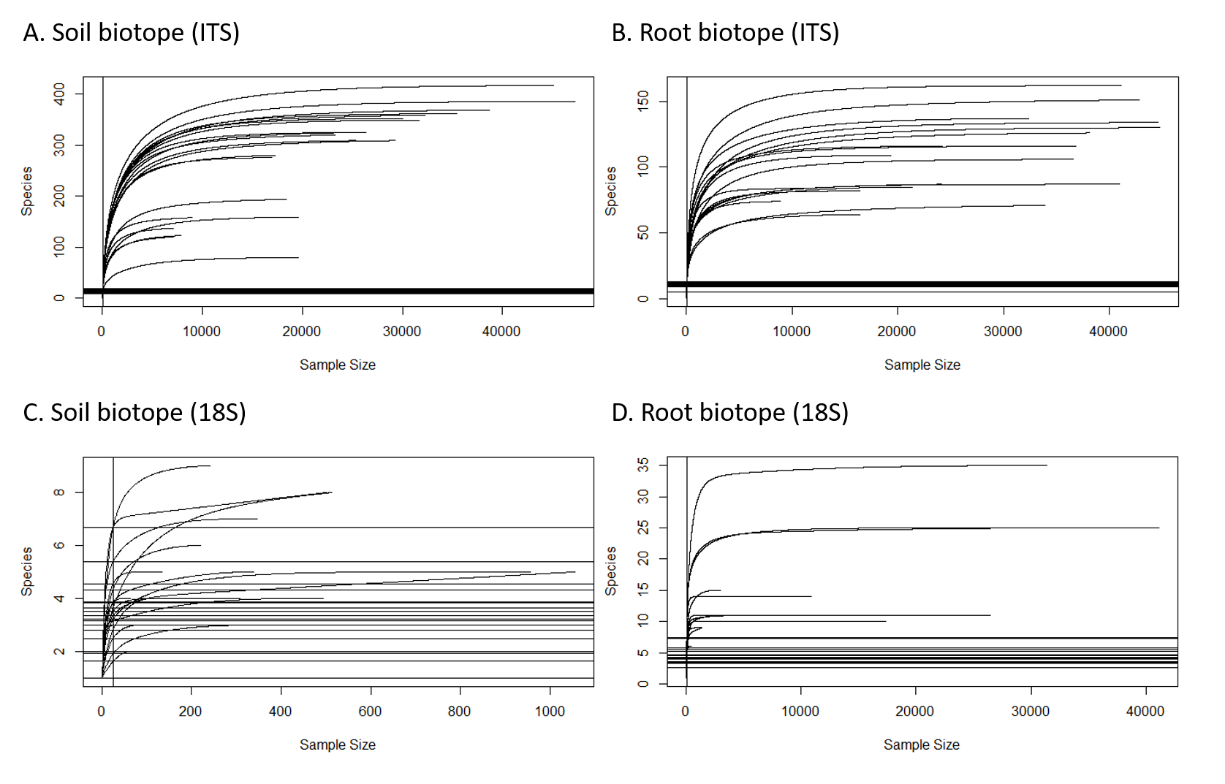


**Figure S1.** Rarefaction curves obtained for the ITS marker (fungi - **A**. and **B**.) and for the 18S rRNA gene (AMF - **C**. and **D**.), for the soil (**A**. and **C**.) and root (**C**. and **D**.) biotopes, involving respectively 25 and 20 samples.


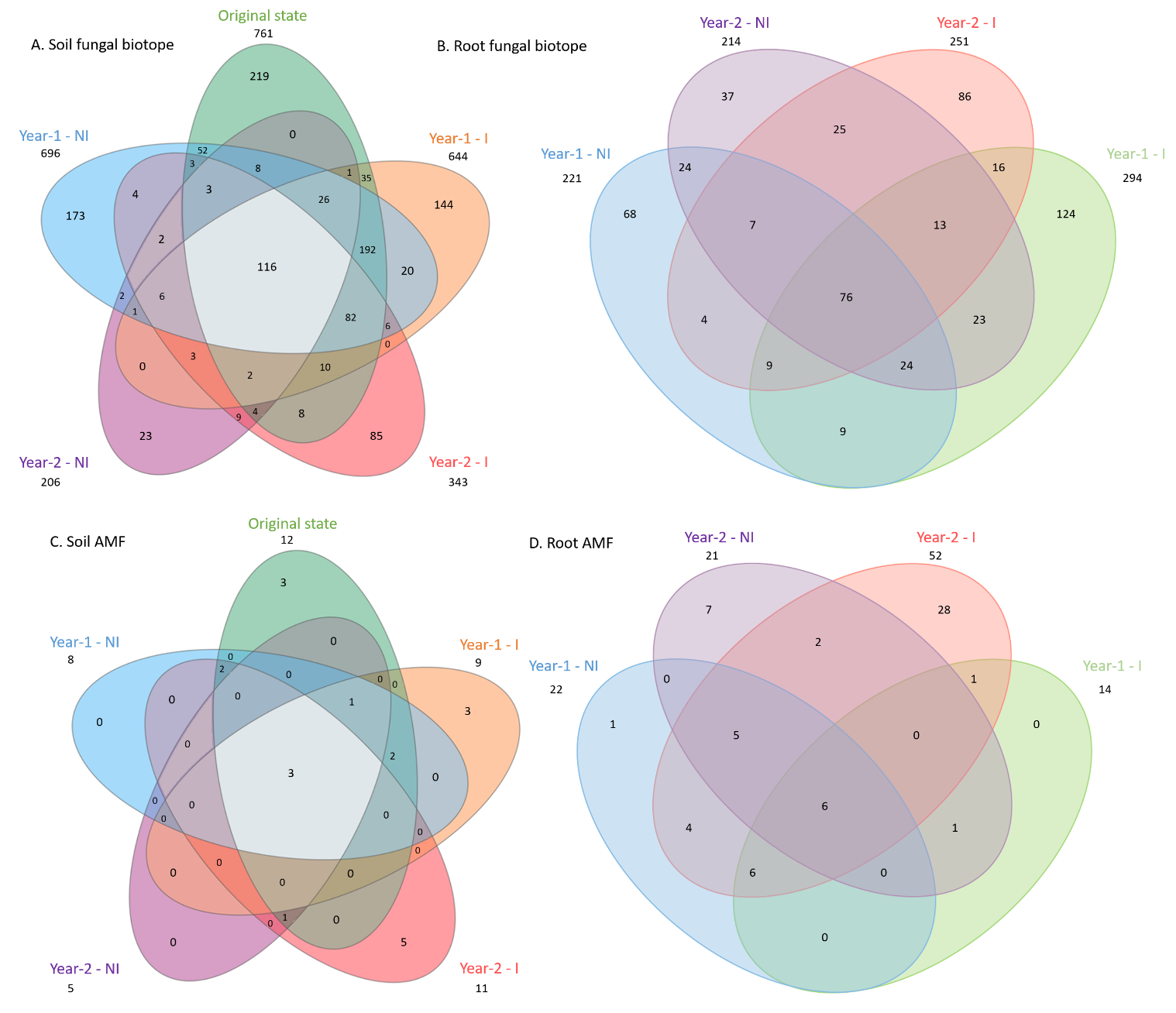


**Figure S2.** Venn diagrams showing the overlap of the fungal (ITS dataset – a. and b. and 18S dataset – c. and d.) community overtime for soil (**A**. and **C**.) and root (**B**. and **D**.) biotopes. These diagrams display the number of total (out of the shapes), specific and shared ASVs. The total numbers of fungal ASVs for the ITS and 18S datasets are respectively 1239 and 20 in soil and 569 and 61 in roots. NI: non-inoculated; I: inoculated.





**Figure S3.** RaxML phylogenetic tree showing the taxonomic assignment of each 18S ASVs to the genus level. The scale represents the branch length corresponding to the expected number of substitutions per site. ASV-R: indicates AMF ASVs identified in the roots; ASV-S: indicates AMF ASVs identified in the rhizospheric soil.


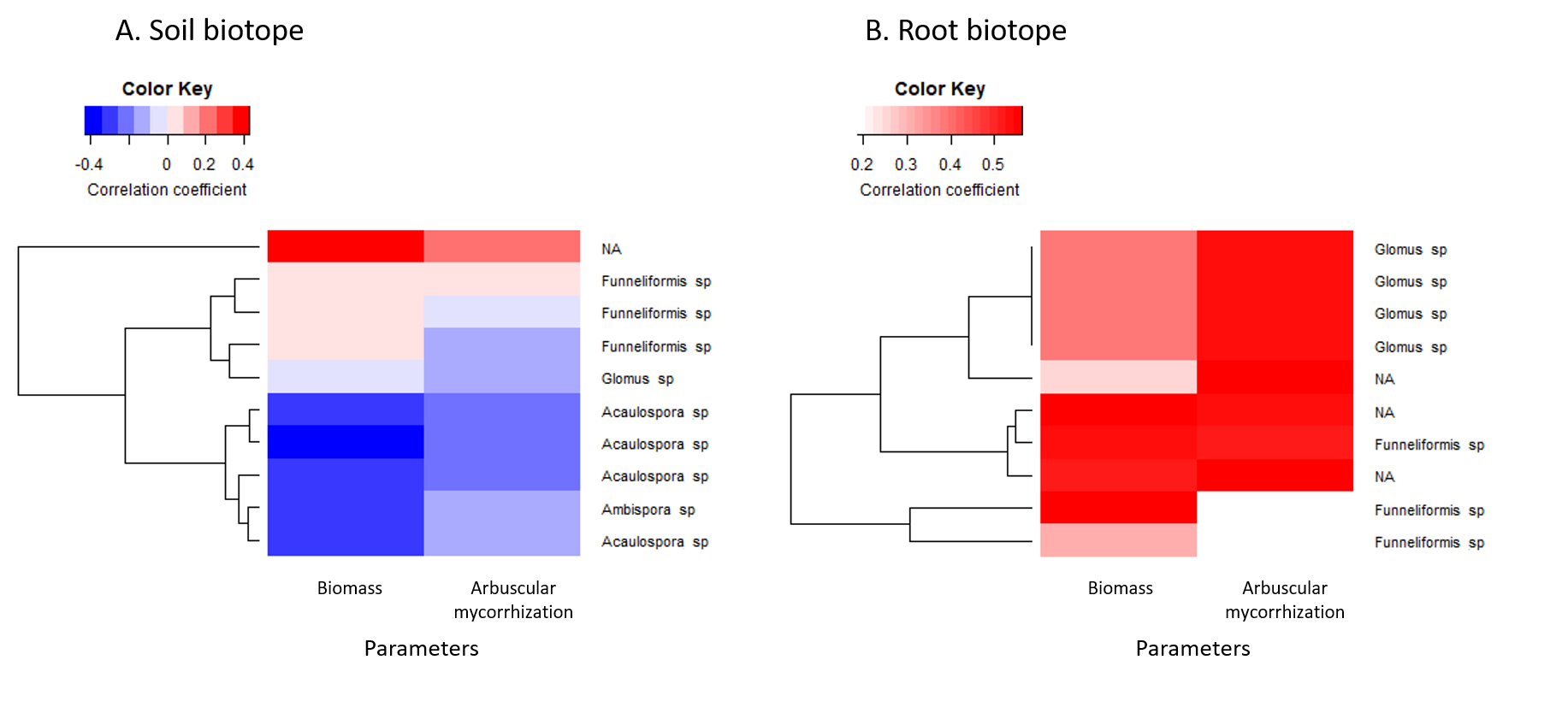


**Figure S4.** Heatmap representation based on the correlation coefficients between clary sage height or arbuscular mycorrhization and the 10 most represented ASVs from the 18S rRNA gene dataset in soil (**A**.) and root (**B**.) biotopes respectively. NA: non-assigned taxonomy.
