# Supplementary material for: Clary Sage Cultivation and Mycorrhizal Inoculation Influence the Rhizosphere Fungal Community of an Aged Trace-Element Polluted Soil
Source: Microorganisms. 2021 Jun 19;9(6):1333. doi: 10.3390/microorganisms9061333 (PMC8234821; doi:10.3390/microorganisms9061333)
Supplement: Supplementary file 1 [file Figure S3.pdf › Figure S3.pdf]

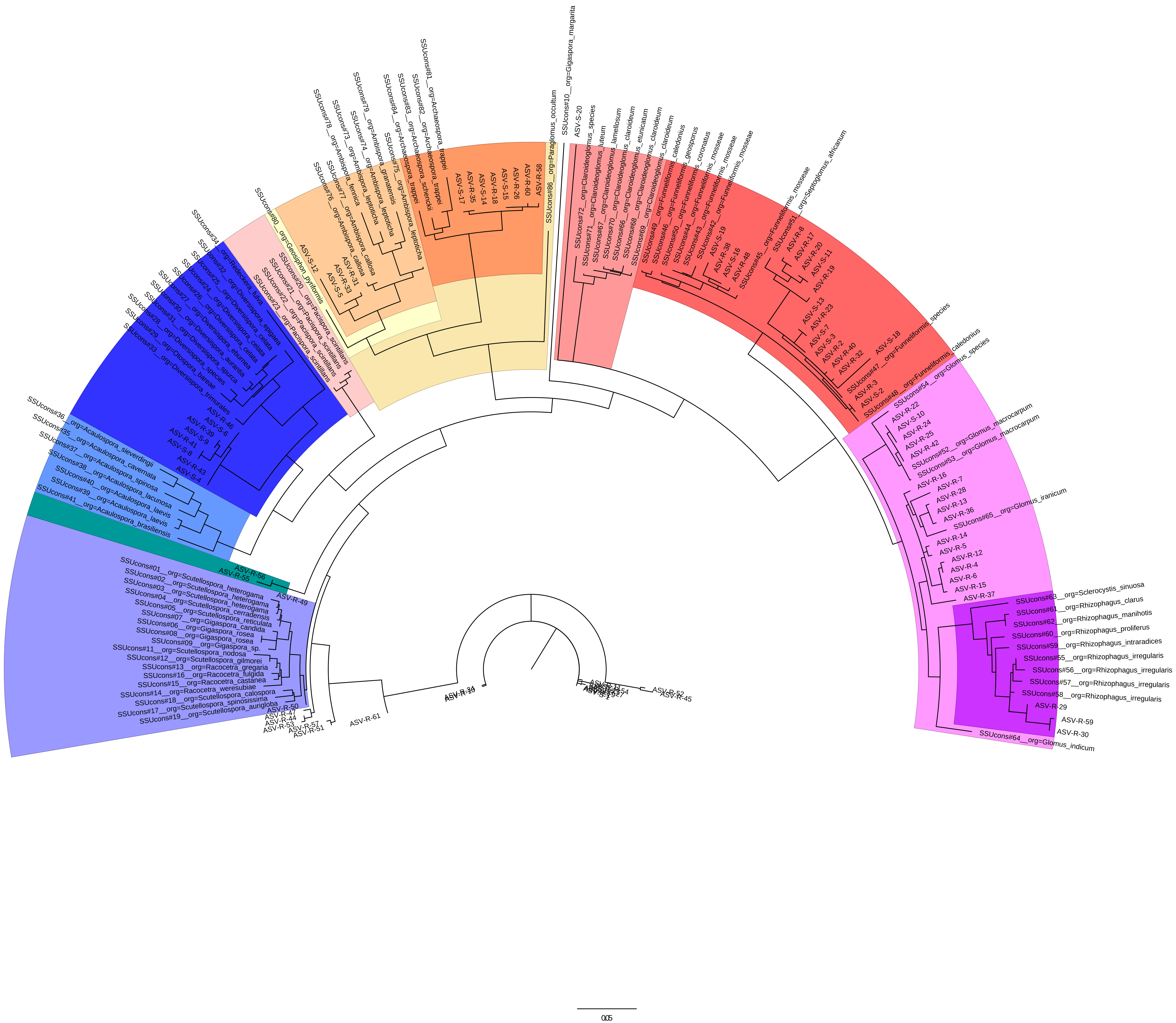
SSUcons#63__org=Sclerocystis_sinuosa
SSUcons#61__org=Rhizophagus_clarus
SSUcons#62__org=Rhizophagus_manihotis
SSUcons#60__org=Rhizophagus_proliferus
SSUcons#59__org=Rhizophagus_intraradices
SSUcons#55__org=Rhizophagus_irregularis
SSUcons#56__org=Rhizophagus_irregularis
SSUcons#57__org=Rhizophagus_irregularis
SSUcons#58__org=Rhizophagus_irregularis
ASV-R-29
ASV-R-59
ASV-R-30
SSUcons#64__org=Glomus_indicum
SSUcons#65__org=Glomus_iranicum
SSUcons#52__org=Glomus_macrocarpum
SSUcons#53__org=Glomus_macrocarpum
SSUcons#01__org=Scutellospora_heterogama
SSUcons#02__org=Scutellospora_heterogama
SSUcons#03__org=Scutellospora_heterogama
SSUcons#04__org=Scutellospora_cerradensis
SSUcons#05__org=Scutellospora_reticulata
SSUcons#07__org=Gigaspora_candida
SSUcons#06__org=Gigaspora_rosea
SSUcons#08__org=Gigaspora_rosea
SSUcons#09__org=Gigaspora_sp.
SSUcons#11__org=Scutellospora_nodosa
SSUcons#12__org=Scutellospora_gilmorei
SSUcons#13__org=Racocetra_gregaria
SSUcons#16__org=Racocetra_fulgida
SSUcons#15__org=Racocetra_castanea
SSUcons#14__org=Racocetra_weresubiae
SSUcons#18__org=Scutellospora_calospora
SSUcons#17__org=Scutellospora_spinosissima
SSUcons#19__org=Scutellospora_aurigloba
SSUcons#36__org=Acaulospora_sieverdingii
SSUcons#35__org=Acaulospora_cavernata
SSUcons#37__org=Acaulospora_spinosa
SSUcons#38__org=Acaulospora_lacunosa
SSUcons#40__org=Acaulospora_laevis
SSUcons#39__org=Acaulospora_laevis
SSUcons#41__org=Acaulospora_brasiliensis
SSUcons#10__org=Gigaspora_margarita
SSUcons#86__org=Paraglomus_occultum
ASV-R-61
